# *In vitro* effect of amorphous calcium phosphate paste applied for extended periods of time on enamel remineralization

**DOI:** 10.1590/1678-7757-2016-0513

**Published:** 2017

**Authors:** Ana Elisa de Mello Vieira, Marcelle Danelon, Danielle Mendes da Camara, Eliana Rodrigues Rosselli, Stuart R Stock, Mark L Cannon, Xianghui Xiao, Francesco de Carlo, Alberto Carlos Botazzo Delbem

**Affiliations:** 1 Univ. Estadual Paulista, Faculdade de Odontologia de Araçatuba, Departamento de Odontologia Infantil e Social, Araçatuba, SP, Brasil; 2 Northwestern University, Feinberg School of Medicine, Department of Molecular Pharmacology and Biological Chemistry, Chicago, IL USA; 3 Northwestern University, Feinberg School of Medicine, Ann and Robert Lurie Children's Hospital, Chicago, IL, USA; 4 Argonne National Laboratory, Advanced Photon Source, Argonne, IL, USA

**Keywords:** X-ray microtomography, Tooth remineralization, Calcium phosphates, Fluoride, Dental enamel

## Abstract

Dental applications based on the unique characteristics of amorphous calcium phosphate stabilized by casein phosphopeptides (CPP-ACP) have been proposed, as well as the improvement of its properties.

**Objectives:**

The objective of this study was to determine the ability of topically applied CPP-ACP from a commercial product to remineralize subsurface lesions when applied for extended periods of time (3 h and 8 h).

**Material and Methods:**

Artificially induced carious lesions were produced in 50 bovine enamel blocks previously selected by surface hardness. After treatments with gel without F and CPP-ACP applied for 1 minute (Placebo); 2% NaF neutral gel applied for 1 minute (Fluoride 1 min); CPP-ACP applied for 3 min (ACP 3 min); and CPP-ACP applied for 3 h (ACP 3 h) and for 8 h (ACP 8 h), the enamel blocks were submitted to the remineralization pH-cycling. Surface hardness and synchrotron micro-tomography were used to determine the percentage of surface hardness recovery (%SHR) and to calculate mineral concentration (g_HAp_.cm^−3^), respectively. The data were submitted to ANOVA followed by the Student-Newman-Keuls test (p<0.05).

**Results:**

Fluoride gel presented higher %SHR followed by ACP 3 min (p<0.001). No difference (p = 0.148) was found for Placebo, ACP 3 h and ACP 8 h groups for %SHR. Fluoride gel showed greater mineral concentration (p<0.001) when compared with the other groups. ACP 3 min demonstrated a significant difference (p<0.001) from ACP 3 h and ACP 8 h. The ACP 3 h and 8 h presented a subsurface lesion with development of laminations in all blocks.

**Conclusion:**

In this *in vitro* study the use of CPP-ACP for extended periods of time did not produce an additive effect in the remineralization process.

## Introduction

Dental applications based on the unique characteristics of casein phosphopeptide-amorphous calcium phosphate (CPP-ACP) have been proposed, as well as the improvement of its properties and associations with similar products that have antidemineralizing and remineralizing potential. The developed systems use a special milk-derived protein (casein phosphopeptides; CPP) to stabilize the calcium phosphate ions from amorphous calcium phosphate (ACP)^4^. The CPP-ACP may then act as a calcium phosphate reservoir, buffering the free calcium and phosphate ion activities, thereby helping to maintain a state of supersaturation with respect to tooth mineral depressing enamel demineralization and enhancing remineralization^12^.

CPP-ACP has been incorporated into various products in order to exert a topical effect. These products include commercially available sugar-free chewing gum, mints and topical gels^12^. The indications for use are during bleaching, following professional tooth cleaning, after application of topical fluoride, for caries active patients, and those suffering from erosion or xerostomia. In view of its broad methods of application for dental care, the CPP-ACP can be used safely from infants through the elderly^12^.

The application of the pastes containing CPP-ACP as a home-care product is commercially available. CPP-ACP has been used as re-mineralizing agent in caries active patients, dentin sensitivity, post bleaching sensitivity, and for tooth erosion^12^ ^,^ ^20^. For these patients, the extended topical application of CPP-ACP may bring a greater reduction in the severity of these dental problems. Since the CPP-ACP formulation is non-toxic^12^ it could be swallowed instead of spat out, allowing the overnight use in a tray by the patient, mainly after dental bleaching. Nevertheless, there is no published research using CPP-ACP with different approaches such as a longer treatment time^15^. Therefore, further research is required to provide a scientifically supported recommendation for longer clinical applications.

Faced with the necessity to provide a scientifically supported clinical recommendation for its use, the aim of this *in vitro* study was to determine the ability of a commercial product containing CPP-ACP to remineralize subsurface lesions when applied for extended periods of time (3 h and 8 h). The null hypothesis was that the product with CPP-ACP applied for 3 minutes would present the same ability to produce remineralization when applied for 3 h or 8 h.

## Material and Methods

### Experimental design

Enamel blocks (4×4 mm) were obtained from bovine incisor teeth that were stored in 2% formaldehyde solution with a pH of 7.0 for 30 days^7^. The enamel surface of the blocks was then serially polished, followed by their selection through surface hardness analysis (SH_0_; 330 up to 376 KHN; p=0.119). The blocks were then demineralized and submitted to post demineralization surface hardness analysis (SH_1_). The blocks were randomly allocated to 5 groups of 10 blocks each: Placebo - gel without F and CPP-ACP applied for 1 minute; Fluoride 1 min - application of neutral F gel (2% NaF, Vigodent, Rio de Janeiro, RJ, Brasil) for 1 minute^7^; ACP 3 min - application of CPP-ACP (MI Paste, GC America Inc, Alsip, IL, USA) for 3 minutes; ACP 3 h - application of CPP-ACP for 3 h; and ACP 8 h - application of CPP-ACP for 8 h. The blocks were rinsed with deionized water jet for 30 seconds and kept in non-stimulated human saliva (2 ml/block) for 30 minutes prior to the pH cycling. Then, the blocks were submitted to pH cycling at 37°C for 6 days^19^. Surface hardness was determined (SH_2_) and the blocks were submitted to synchrotron micro-computed tomography (SMCT) analysis, to calculate the integrated loss of subsurface mineral and analyze the lesion depth (μm). The variation factors were the materials and the variables were SH_2_, Δg_HAp_.cm^−3^, thickness of enamel surface layer and lesion depth (μm).

### Products formulations

The gels had the following ingredients: glycerol, propylene glycol, hydroxyethyl cellulose, sodium methyl-p-hydroxybenzoate, sodium saccharin, flavoring, pigment and pure water (pH 6.8). Fluoride gel also contained 2% of sodium fluoride (2% NaF, Vigodent, Rio de Janeiro, RJ, Brasil). The MI Paste was formulated with: casein phosphopeptides and amorphous calcium phosphate, glycerol, D-sorbitol, sodium carboxymethyl cellulose, silicon dioxide, titanium dioxide, xylitol, phosphoric acid, flavoring, zinc oxide, sodium saccharin, ethyl p-hydroxybenzoate, magnesium oxide, guar gum, propyl p-hydroxybenzoate, butyl p-hydroxybenzoate, water and pH 7.8 (MI Paste, GC America Inc, Alsip, IL, USA).

### Subsurface enamel demineralization

The surface of each specimen, except enamel, was coated with acid-resistant varnish and subsurface enamel demineralization was produced, after SH_0_, by immersing each enamel block in 32 mL of a solution with 1.3 mmol/l Ca, 0.78 mmol/l P in 0.05 mol/l acetate buffer, pH 5.0; 0.03 ppm F; for 16 h at 37°C^16^ ^,^ ^18^. The surface hardness of the enamel blocks was measured again (SH_1_). Mean (SD) of SH_1_ was 62.7 (24.7) KHN; the lowest mean value was 56.5 (25.1) and the highest was 68.0 (26.7) (p=0.946).

### pH-cycling of remineralization

The effect of products promoting the remineralization of carious lesions was evaluated using the model of Vieira, et al.^19^ (2005). The surface of each specimen, except enamel, was coated with acid-resistant varnish and the blocks were individually submitted to pH cycling, at 37°C for 6 days. The remineralizing solution (1.5 mmol/l Ca, 0.9 mmol/l P, 0.15 mmol/l KCl, 0.02 mol/l cacodylate buffer, 0.02 ppm F, pH 7.0; 4 ml/block) was changed twice a day (8 a.m. and 4 p.m.). The cariogenic challenges were achieved using a demineralizing solution (2.0 mmol/l Ca, 2.0 mmol/l P, 0.075 mol/l acetate buffer, 0.03 ppm F, pH 4.7; 12 ml/block) for two hours (12 p.m. to 2 p.m.), which was freshly changed once a day. The blocks were rinsed with deionized water at each change of solution.

### Analysis of surface hardness

The hardness (KHN) of the enamel surface (SH) was determined using a Shimadzu HMV-2000 microhardness tester (Shimadzu Corp., Kyoto, Kyoto, Japan) and a Knoop diamond under a 25 g load for 10 s. Five indentations spaced 100 pm from one another were made at the center of the enamel surface (SH_0_). Indentations for SH_1_ and for SH_2_ were spaced 100 μm from one another and from the baseline. The percentage of surface hardness recovery (%SHR) was then calculated [%SHR=((SH_2_-SH_1_)/(SH_0_-SH_1_))x100].

### Analysis of synchrotron micro-computed tomography

Six blocks of each group were sectioned longitudinally and the samples (2×4 mm) were submitted to synchrotron micro-computed tomography (SMCT), at bending magnet station 2-BM of Advanced Photon Source (APS), Argonne National Laboratory, Argonne, IL, USA. X-ray photons with energy of 20 keV were provided by a double multi-layer monochromator (DMM)^2^. The detector system consisted of a 12-bit, 2K x 2K CCD camera coupled with an optical lens (2.5X) to a CdWO_4_ single-crystal phosphor. Views were recorded every 0.25° from 0° to 180° and were normalized for detector and beam non-uniformities. The specimens were reconstructed on a 2K x 2K grid of isotropic voxels, side length ~2.8 μm. The analysis was based on mineral concentrations calculated from the linear attenuation coefficient (p) and described as mass of pure hydroxyapatite (p=3.15 g×cm^−3^) *per* unit volume of tissue (g_HAp_.cm^−3^)^9^ ^,^ ^10^.

The analysis was performed up to 221.2 μm below the surface and the mean value of mineral concentration (g_HAp_.cm^−3^) was determined for each unit of depth (2.8 μm) in each slice. Three areas in the central region of the slices were analyzed using Software Image J to determine the lesion depth (μm). The integrated area under the curve (cross-sectional mineral profiles into the enamel) was calculated, using mineral concentration values (g_HAp_.cm^−3^), by the trapezoidal rule (GraphPad Prism, version 3.02, GraphPad Software Inc., La Jolla, CA, USA) for each depth (μm) from the lesion to sound enamel. This value was subtracted from the integrated area of sound enamel to obtain the integrated area of subsurface regions in enamel, which was named integrated loss of subsurface mineral (IML, g_HAp_.cm^−3^ .μm)^8^.

To analyze the patterns of remineralization in relation to placebo, differential mineral concentration profiles were calculated by subtracting the mineral values of each group at each depth from the Placebo group (i.e., Fluoride 1 min, ACP 3 min, ACP 3 h and ACP 8 h groups values minus the Placebo group). These differential profiles were then integrated over two depth zones in the lesion (zone A: 2.8-16.8 μm and zone B: 19.6-33.6 μm) and underlying sound enamel to yield ΔIML values^5^ ^,^ ^6^.

### Statistical analysis

Analyses were performed using the SigmaPlot software (version 12.0, Systat Software Inc., San Jose, CA, USA) and the level of statistical significance was established at 5%. Data from SH_2_, %SHR, IML and lesion depth showed normal and homogeneous distributions and were submitted to ANOVA (One-way) followed by Student-Newman-Keuls test. The ΔIML values were submitted to two-way ANOVA followed by the Student-Newman-Keuls test. Pearson's correlation coefficient was calculated for SH_2_ and IML, and SH_2_ and lesion depth.

## Results

The Fluoride 1 min group presented the highest mean value for SH_2_ and %SHR (Table 1) compared with the other groups (p<0.001). The Placebo, ACP 3 h and ACP 8 h showed similar results (SH_2_: p=0.599; %SHR: p=0.148) with lower values compared with the other groups (p<0.001).

**Table 1:** Mean values (SD) of hardness (n=10) and synchrotron (n=6) enamel analysis according to the treatments

Treatments	Variables			
	SH_2_ (KHN)	%SHR	IML (g_HAp_.cm^−3^ x μm)	Lesion Depth (μm)
Placebo	115.8^a^	18.1^a^	-13.1^a^	82.6^a^
	(32.6)	(5.1)	(4.4)	(5.8)
Fluoride 1 min	200.6^b^	46.4^b^	-1.4^b^	40.1^b^
	(21.2)	(4.4)	(0.9)	(9.6)
ACP 3 min	143.1^c^	25.3^c^	-4.3^c^	41.1^b^
	(23.9)	(4.6)	(15)	(2.3)
ACP 3 h	102.1^a^	13.3^a^	-17.5^e^	78.4^a^
	(34.9)	(7.1)	(3.1)	(13.5)
ACP 8 h	107.7^a^	14.1^a^	-11.3^a^	72.8^a^
	(23.6)	(4.7)	(5.2)	(8.7)

Lowercase letters indicate statistically significant differences (Student-Newman-Keuls; p<0.05) between the groups in each analysis.

SH_2_=surface hardness after pH-cycling.

%SHR=percentage of surface hardness recovery.

IML=integrated loss of subsurface mineral.

The mean (SD) mineral concentration value for sound bovine enamel of the blocks analyzed was 2.47 (0.03) g_HAp_.cm^−3^ (2.40-2.53). The results from the SMCT analysis showed that the Fluoride 1 min group presented the lowest value (p<0.001) for integrated loss of subsurface mineral (IML) compared with the other groups (Table 1). There was a statistically significant difference (p<0.001) between the ACP 3 min and the ACP 3 h and ACP 8 h groups (Table 1). There was also a significant positive correlation between SH_2_ and IML (r value=0.839; R^2^=0.704).

**Figure 1:**
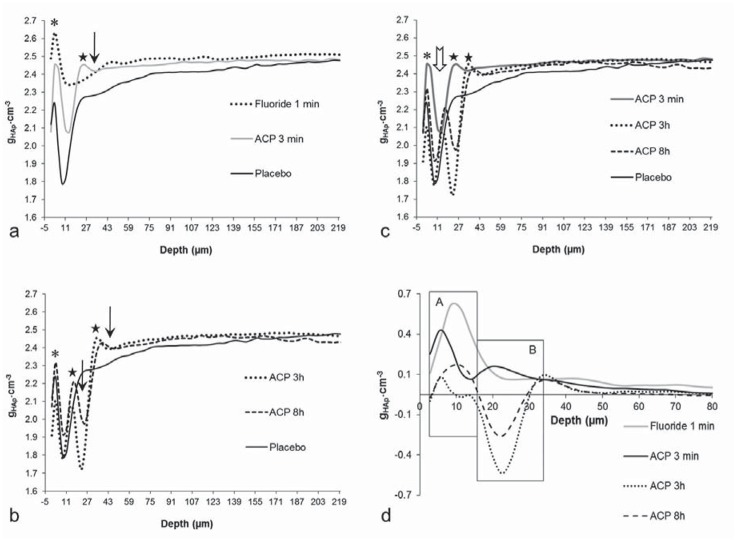
Depth profiles of mineral concentration (g_HAp_.cm^−3^) in lesions for each treatment, (a) Comparison of mineral profiles from Placebo, Fluoride 1 min and ACP 3 min groups, (b) Mineral profiles from Placebo, ACP 3 h and ACP 8 h. (c) Mineral profiles from Placebo, ACP 3 min, ACP 3 h and ACP 8 h. (d) Differential mineral concentration profiles as a function of depth according to the treatments: zone A: 2.8-16.8 μm and zone B: 19.6-33.6 μm. *: maximum mineral concentration on outer enamel layer. ★: maximum mineral concentration through the lesion for each group. A: subsurface lesion. ↓: lamination

The lesion depth (Table 1 and Figure 1) was higher in the placebo and ACP 3 h and ACP 8 h groups (p=0.126) when compared with the groups treated with fluoride or ACP (p<0.001). ACP 3 min and Fluoride 1 min groups presented similar (p = 0.849) depth values (Table 1). There was a significant negative correlation between the lesion depth and SH_2_ (r value=-0.846, R^2^=0.716).

**Figure 2:**
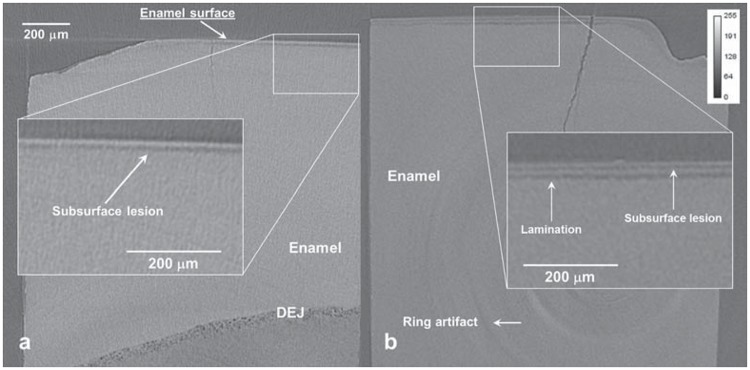
Synchrotron micro-computed tomography (SMCT) slice from a typical specimen of the (a) placebo and (b) ACP 8 h group. The lighter the voxel, the higher the mineral concentration. The dentino-enamel junction is labeled DEJ

The mineral concentration profile in function of the depth demonstrated an outer enamel hypermineralization for Fluoride 1 min group (Figure 1a and 1d). A well-defined superficial enamel layer was followed by an area of low mineralization in all groups (Figure 1 and Figure 2a). The ACP 3 h and ACP 8 h groups presented a subsurface lesion with development of laminations in all blocks of the two groups (Figure 1b and 1c, Figure 2b).

The values integrated from differential mineral profile provided evidence of different mineral recovery patterns (Table 2 and Figure 1d). The remineralization process for Fluoride 1 min group was ~74% lower at zone B (19.6-33.6 μm) when compared with zone A (2.8-16.8 μm) (p<0.001). This relation was ~40% for ACP group (p<0.019). There was different mineral loss at zone B for ACP 3 h and ACP 8 h groups compared with the treatments of the other groups (p<0.001).

**Table 2:** Mean values (SD) of integrated of differential profiles (ΔIML) calculated for two zones (Figure 1d) in the enamel lesions according to the treatments

Treatments	ΔIML (g_HAp_.cm^−3^ x μm)	
	zone A (2.8-16.8 μm)	zone B (19.6-33.6 μm)
Fluoride 1 min	6.24^a,A^	1.60^a,B^
	(0.69)	(0.40)
ACP 3 min	3.56^b,A^	2.11^a,B^
	(0.79)	(0.61)
ACP 3 h	0.08^d,A^	-3.50^b,B^
	(143)	(166)
ACP 8 h	1.71^c,A^	-1.59^c,B^
	(2.29)	(2.47)

Lowercase letters indicate statistically significant differences between the groups in each zone. Capital letters indicate the differences between zone A and zone B for each treatment (Student-Newman-Keuls; p<0.05).

Although ring artifacts were observed in all reconstructed images, they did not interfere with the measurements of mineral concentrations in enamel up to 221.2 μm of depth, as they were closer to the dentino-enamel junction (Figure 2).

## Discussion

The present *in vitro* laboratory study evaluated the effectiveness of CPP-ACP, as it would be applied for extended time in trays. As a null hypothesis, the CPP-ACP applied for 3 minutes would present the same remineralizing effect as compared with CPP-ACP applications for 3 hours or 8 hours. The ACP 3 h group was tested because it is stated by the manufacturer (instructions for use by GC Corporation, Tokyo, Japan) that the longer the CPP-ACP stays in the mouth, the more effective it will be releasing calcium and phosphate continuously for the 3 hours. As there is the necessity of more research using CPP-ACP with different approaches, such as longer treatment time^15^, the time of 8 hours was tested for comparisons. Clinically, the patient would be instructed to apply the paste using a tray for 3 or 8 hours (overnight), mainly after dental bleaching.

Application of CPP-ACP for 3 minutes (ACP 3 min) was capable of improving enamel remineralization (Table 1,Table 2 and Figure 1), reducing mineral loss (IML and ΔIML) and lesion depth (μm). As described in the literature, the CPP-ACP has the ability to adhere to enamel and supersaturate the environment with free calcium and phosphate ion activities^3^ ^,^ ^11^ ^,^ ^17^. Both the outer part and the inner part of the lesion showed a more homogeneous remineralization ratio (Table 2). Probably the great diffusion of calcium phosphate neutral ion pair (CaHPO_4_^0^), produced by calcium and phosphate supersaturation^1^ ^,^ ^11^, leads to a higher mineral recovery in the inner part of the lesion (zone B). The diffusion of charged ions (Ca^2+^ and PO_4_^3-^) through a charged enamel surface layer is lower than neutral ion^2^. Despite producing lower mineral recovery than the Fluoride 1 min group, the greater diffusion of neutral calcium phosphate reduced the lesion depth at the same level (Table 1 and Figure 1).

In the presence of fluoride the effect is higher in the outer part of the lesion (zone A, Table 2 and Figure 1a) mainly due its strong deposition into the outer zones of the enamel lesion^13^ ^,^ ^14^. In addition, the presence of fluoride promoted higher ionic activity to HF^0^ and greater degree of saturation with respect to fluorapatite that promote a higher impact on remineralization^11^. The precipitation of fluoride products in the outer part of enamel resulted in higher surface hardness (Table 1) and mineral content (zone A, Table 2) than the products precipitated by CPP-ACP. Surface hardness varied according to the integrated loss of subsurface mineral (IML) and lesion depth, allowing an evaluation of mineral loss through a faster and more direct method. However, it did not identify the type of mineral profile in lesions for each treatment (Figure 1).

The increase in the time of contact with the surface enamel (3 h and 8 h) simulating an overnight application did not show superior mineral gain compared with the placebo group as well as for lesion depth. It had been expected that by maintaining prolonged contact of CPP-ACP with enamel would result in wider diffusion of neutral calcium phosphate into the enamel reducing the lesion to a greater extent. The greater time of contact with enamel produced laminations in the extension of the original lesion with mineral deposition within the body lesion (Figure 1b and 1c). The development of lamination is due to alternating de-/remineralization episodes and has been observed in the presence of fluoride that can be adsorbed in varying concentrations at various depths, resulting in differences in acid susceptibility of the mineral^13^ ^,^ ^14^. However, there is no previous report on the development of lamination produced by supersaturation of calcium and phosphate from a source of stabilized casein phosphopeptide-amorphous calcium phosphate (CPP-ACP). But the zones of high and low mineral content through the lesion were similar to those described by Lagerweij and ten Cate^13^ (2006): the surface zone with high mineral concentration, the original lesion with low mineral concentration, followed by a zone with a high mineral density, then a secondary lesion which is formed during the short demineralization of the pH-cycling model, and underneath these lesion zones is the sound enamel (Figure 1a, 1b and 1c).

The prolonged contact with the enamel leads to a great precipitation of calcium phosphate on enamel surface from CPP-ACP with formation of laminations through the lesion during the pH cycling process (Figure 1b, 1c and Figure 2). With the development of laminations, it seems likely that the mineral precipitated from CPP-ACP is not capable of reducing the acid diffusion through the lamination, increasing the mineral loss in the periphery of the lesion. It is possible that the higher precipitation of CPP-ACP led to obstruction of the enamel pores, hindering CaHPO_4_^0^ diffusion into the enamel lesion. Nevertheless, the precipitate from CPP-ACP does not prevent the acid diffusion and it still occurs into the enamel producing a new area of demineralization beyond the inner boundary of the outermost demineralized area (Figure 1b, 1c and Figure 2). As the ionic exchanges with the medium are altered due to pore obstruction, part of the demineralization product accumulates in the boundary between the outermost and innermost demineralized areas, forming a zone with greater mineral density. This may explain in part the lower remineralization capacity of CPP-ACP applied for 3 minutes in relation to fluoride gel. There is no increase in lesion depth due to the short time in acid medium. Thus, the lower resistance of mineral precipitated from the CPP-ACP applied for extended time to acid dissolution and diffusion presented the formation of laminations. The mineral (re)precipitation produced in the presence of fluoride presented greater capacity to hinder the acid diffusion and presented lower acid dissolution in the remineralization model with the short time of demineralization. It is possible to observe in the region between 50.4 to 61.6 μm a remnant of lamination in the fluoride group (Figure 1a).

## Conclusion

Based on the results of this *in vitro* study, the use of CPP-ACP for extended periods of time as a way of increasing the effectiveness of the product produced no additive effect on the remineralization process. Additional studies should be performed using MI Paste plus as it also contains 850 ppm fluoride.
